# Targeted ErbB4 receptor activation prevents D-galactose-induced neuronal senescence via inhibiting ferroptosis pathway

**DOI:** 10.3389/fphar.2025.1528604

**Published:** 2025-01-31

**Authors:** Ji-Ji Dao, Wei Zhang, Chong Liu, Qian Li, Chen-Meng Qiao, Chun Cui, Yan-Qin Shen, Shuang-Xi Chen, Wei-Jiang Zhao

**Affiliations:** ^1^ Cell Biology Department, Wuxi School of Medicine, Jiangnan University, Wuxi, Jiangsu, China; ^2^ MOE Medical Basic Research Innovation Center for Gut Microbiota and Chronic Diseases, Wuxi School of Medicine, Jiangnan University, Wuxi, Jiangsu, China; ^3^ Department of Pathogen Biology, Guizhou Nursing Vocational College, Guiyang, Guizhou, China; ^4^ Laboratory of Neurodegenerative and Neuroinjury Diseases, Wuxi School of Medicine, Jiangnan University, Wuxi, Jiangsu, China; ^5^ The First Affiliated Hospital, Department of Neurology, Multi-Omics Research Center for Brain Disorders, Hengyang Medical School, University of South China, Hengyang, Hunan, China; ^6^ Clinical Research Center for Immune-Related Encephalopathy in Hunan Province, Hengyang Medical School, University of South China, Hengyang, Hunan, China

**Keywords:** ErbB4 receptor, small molecule, senescence, D-galactose, hippocampus, neuron, ferroptosis, neurodegenerative diseases

## Abstract

**Background:**

Neuronal senescence is a common pathological feature of various neurodegenerative diseases, with ferroptosis playing a significant role. This study aims to investigate the role of ErbB4 receptor activation in preventing D-Galactose (D-gal)-induced neuronal senescence.

**Methods:**

Mice subjected to D-gal-induced aging were administered a small molecule ErbB4 receptor agonist (E4A), identified via virtual screening, melatonin, or a combination of both. Behavioral assessments were conducted to evaluate therapeutic efficacy in memory and cognitive functions. Immunofluorescence staining, western blot, and biochemical assays were primarily employed to assess changes in both senescence- and ferroptosis-related molecules in mouse hippocampal tissues in response to each treatment. Additionally, mouse hippocampal HT22 neuronal cell cultures were utilized to corroborate the in vivo findings.

**Results:**

The targeted activation of ErbB4 receptor by E4A significantly ameliorated the behavioral deficits induced by D-gal in mice, demonstrating an effect comparable to that of melatonin, a natural inhibitor of *in vivo* senescence and ferroptosis. Both E4A and melatonin mitigated D-gal-induced aging in hippocampal neurons of mice. This was evidenced by the upregulation of Lamin B1 and the downregulation of P53, P21, P16, GFAP, and Iba-1 expression levels. Moreover, D-gal treatment markedly decreased the protein expression of the ferroptosis inhibitor Nrf2 while augmenting the expression of the ferroptosis promoter TFRC. These alterations were partially reversed by the individual administration of E4A and melatonin. *In vitro* studies further corroborated that D-gal treatment significantly and concurrently induced the expression of senescence markers and ferroptosis promoters. However, both E4A and melatonin were able to significantly reverse these changes. Additionally, E4A markedly ameliorated Erastin-induced ferroptosis in mouse hippocampal neuronal cells.

**Conlusion:**

Our findings suggest that targeted activation of ErbB4 receptor may be a viable strategy for treating neuronal senescence by inhibiting ferroptosis, thereby offering a potential therapeutic avenue for senescence-associated neurodegenerative diseases.

## 1 Introduction

With the escalating trend of global aging, the exploration of strategies for the prevention and management of aging processes and aging-linked illnesses has risen to the forefront of life science discussions. The elderly populace is often besieged by a myriad of chronic health conditions, which not only greatly diminish their quality of life but also significantly amplify the societal burden (Becker et al., 2022). Aging is a pivotal factor in the majority of neurodegenerative diseases, and the process of senescence within the human body can impact all organs, with the brain being among the first to show signs of aging. As the human body progresses through the years, the brain undergoes a series of intricate transformations that gradually affect its functionality (Bishop et al., 2010). Neurodegenerative diseases include Alzheimer’s disease (AD), Parkinson’s disease (PD), Huntington’s disease (HD) and frontotemporal dementia, etc. The common feature of these diseases is the gradual decline, loss and death of the structure and function of neurons, resulting in irreversible neurological dysfunction.

Neurons constitute the fundamental structural and functional units of the nervous system. With advancing age, the neuronal population in the brain diminishes, progressively manifesting a neuronal senescence phenotype. This decline induces a series of dysfunctions, culminating in the onset of age-related neurodegenerative diseases (Ma and Farny, 2023). Empirical research has shown that primary rat hippocampal neurons, when cultured *in vitro* for extended periods, exhibit pronounced senescence-like characteristics. These include elevated β-galactosidase (SA-β-gal) activity and increased expression of the aging marker P16 (Ishikawa and Ishikawa, 2020). Senescent phenotypes of hippocampal neurons have been observed in AD patients (Herdy et al., 2022), characterized by increased p38/MAPK activity and elevated expression of TGF-β mRNA and IL-6 (Si et al., 2021). Additionally, in aged C57BL/6 mice, approximately 40%–80% of Purkinje neurons and 20%–40% of cortical, hippocampal, and peripheral neurons exhibit severe DNA damage, which induces neuronal senescence and contributes to brain aging (Jurk et al., 2012).

Iron, the most abundant transition metal in the human body, is essential for sustaining life; however, studies have shown that iron deposits can accumulate with age, exacerbating adverse effects (Cook and Yu, 1998). In the brain, iron levels increase with aging, and excessive iron deposits disrupt cellular iron homeostasis, inducing ferroptosis-a form of cell death associated with the onset and progression of age-related neurodegenerative diseases (Tian et al., 2022).

Ferroptosis, first identified in 2012, is characterized by iron-dependent lipid peroxidation damage (Jurk et al., 2012). Compared to other forms of cell death, including apoptosis, necrosis, and autophagy, ferroptosis is characterized by distinct mitochondrial alterations such as membrane shrinkage, increased membrane density, blurred cristae, and reduced or absent mitochondrial ridges. Biochemically, ferroptosis is marked by intracellular iron overload and the excessive accumulation of lipid peroxides and reactive oxygen species (ROS) (Friedmann Angeli et al., 2014). Recently, the relationship between iron-mediated cell death and the aging process has attracted considerable interest. A growing body of research indicates that ferroptosis plays a crucial role in the onset and progression of neurodegenerative diseases associated with aging, particularly AD, PD, and amyotrophic lateral sclerosis (ALS) (Mahoney-Sanchez et al., 2021; Wang et al., 2022; Wang et al., 2023). The principal biochemical characteristics of ferroptosis encompass the accumulation of ROS, the depletion of glutathione (GSH), and the diminished activity of glutathione peroxidase 4 (GPX4) (Dixon et al., 2012). Research has demonstrated that GPX4 knockout mice exhibit marked deficits in learning, memory, and cognitive function compared to wild-type mice, which can be ameliorated through the administration of ferroptosis inhibitors (Hambright et al., 2017). Additionally, reduced GSH levels have been observed in patients with AD, where GSH, serving as a cofactor for GPX4, exerts an anti-ferroptotic effect (Ashraf et al., 2020). Furthermore, studies have indicated that the GPX4 content in the substantia nigra of patients with PD is significantly diminished (Bellinger et al., 2011). Therefore, the inhibition of ferroptosis could potentially serve as a strategy to delay aging and mitigate age-related degenerative diseases.

ErbB4, a member of the receptor tyrosine kinase family that includes ErbB1 through ErbB4, is activated by various EGF-like Neuregulin (NRG) proteins (Ou et al., 2021). These proteins are crucial for both developmental processes and the functioning of the mature central nervous system. On the cell surface, ligand-activated ErbB receptors are present in a dimerized form. Dimerization induces a conformational alteration in the receptor, resulting in the phosphorylation of specific tyrosine residues within the cytoplasm. This phosphorylation event facilitates the recruitment of intracellular signaling molecules, consequently activating a cascade of related genes that regulate cellular processes such as growth, differentiation, proliferation, angiogenesis, and apoptosis (Ahsan et al., 2009). Among the ErbB receptors, ErbB4 is unique in its ability to generate intracellular signal transduction through the formation of homodimers and its interaction with NRGs, such as NRG1 (Buonanno and Fischbach, 2001). Currently, it is known that ErbB4 receptors can be activated by binding to seven ligands, including a few ligands in the NRG and EGF families, and NRG1/ErbB4 signaling plays an important role in the nervous system (Buonanno and Fischbach, 2001), and is involved in the occurrence and development of a variety of neurodegenerative diseases. Dysfunction in NRG1/ErbB4 signaling within the hippocampus has been implicated in memory decline in models of LPS-induced systemic inflammation (Gao et al., 2023). Furthermore, NRG1/ErbB4 signaling has been shown to exert a protective effect against Aβ-induced damage to hippocampal long-term potentiation (LTP) (Min et al., 2011).

Recent research has demonstrated that recombinant human NRG1 (rhNRG1) can markedly enhance cardiac function in animal models of heart failure, including ischemic heart disease and viral cardiomyopathy (Fang et al., 2017). Nonetheless, clinical investigations focusing on the treatment of neurodegenerative diseases remain limited. Concurrently, there is a growing body of studies employing targeted molecular docking techniques to identify small molecule compounds with improved central nervous system permeability, aimed at treating neurodegenerative diseases associated with aging (Shinde et al., 2015). In this study, we investigated a small molecule compound targeting conserved amino acid sites of ErbB4 in both humans and mice to assess its potential in delaying D-gal-induced neuronal aging in mice by inhibiting neuronal ferroptosis.

Melatonin, an indole endocrine hormone secreted by the pineal gland, possesses both hydrophilic and lipophilic properties and exerts a wide range of biological functions through binding to three membrane receptors. These functions include the regulation of circadian rhythm and sleep, immune enhancement, metabolic regulation, antioxidant activity, anti-aging effects, and anti-tumor properties (Chen et al., 2020). Recent studies have indicated that melatonin is integral to the pathophysiological mechanisms underlying various neurodegenerative diseases. It exerts its effects by mitigating oxidative stress both *in vivo* and *in vitro* and by reducing the abnormal accumulation of proteins, thereby influencing the progression of these diseases (Li et al., 2020). Specifically, in the context of AD, melatonin has been shown to alleviate amyloid-beta (Aβ) deposition and diminish neuronal loss through its anti-inflammatory and antioxidant properties, ultimately aiding in the restoration of cognitive function (Li et al., 2020). Research has demonstrated that melatonin activates the ERK/Nrf2 signaling pathway through its interaction with MT2 receptors, thereby ameliorating cognitive impairment and hippocampal ferroptosis induced by acute sleep deprivation (Wang et al., 2021). Additionally, other studies have indicated that exogenous melatonin therapy can mitigate pathological changes resulting from hypoxic-ischemic brain injury and inhibit neuronal ferroptosis (Gou et al., 2020). These studies indicate that melatonin may exert anti-aging effects through the regulation of ferroptosis. Melatonin also possesses the ability to modulate the expression of the ErbB4 receptor. Previous research has demonstrated that melatonin can upregulate ErbB4 expression, effectively reduce ROS levels, and thereby enhance antioxidant capacity (Moshkdanian et al., 2017; Shafiei et al., 2023).

In this study, we utilized melatonin as a positive control to investigate the effects of a small molecule agonist targeting ErbB4 receptor activation in the treatment of D-gal-induced neuronal senescence through the inhibition of ferroptosis. We also explored the potential synergistic effects of combining the ErbB4 agonist with melatonin in addressing neuronal senescence.

## 2 Materials and methods

### 2.1 Animals and drug treatment

Fifty healthy male C57BL/6J mice, with an average weight of 18–22 g and aged 8 weeks, were provided by Charles River (Zhejiang, China). All experimental protocols were conducted in accordance with the animal care and usage guidelines. The animal experiments received approval from the Institutional Animal Care and Use Committee of Wuxi School of Medicine, Jiangnan University (Ethics Approval Number: JN.NO20240315c0400701[110]). The ErbB4 receptor agonist, C11H7BrO3 (4-bromo-1-hydroxy-2-naphthoic acid, S987115, Sigma-Aldrich (Shanghai) Trading Co. Ltd., China), which targets the conserved S42, L96, and Y123 sequences within both human and mouse ErbB4 receptor motifs, was previously identified through virtual screening.

The animals were housed in environmentally controlled SPF facilities under a 12-h light/dark cycle at a constant temperature of 22°C, with *ad libitum* access to water and food. Following a one-week adaptation period, the mice were randomly allocated into five experimental groups as follows: (1) Control group: received intraperitoneal (i.p.) administration of normal saline; (2) D-gal group: received 100 mg/kg of D-gal (ST1218, Beyotime) intraperitoneally on a daily basis for 11 weeks; (3) D-gal + E4A group: received 100 mg/kg of D-gal daily for 7 weeks, followed by concurrent i.p. administration of D-gal and 400 ng/kg of E4A daily for 4 weeks; (4) D-gal + MLT group: received 100 mg/kg of D-gal daily for 7 weeks, followed by the concurrent i.p. administration of D-gal and 10 mg/kg of melatonin (MLT, ST1497, Beyotime) daily for 4 weeks; (5) D-gal + E4A + MLT group: received 100 mg/kg of D-gal daily for 7 weeks, followed by concurrent i.p. administration of 100 mg/kg of D-gal, 400 ng/kg of E4A, and 10 mg/kg of melatonin daily for 4 weeks.

### 2.2 Morris water maze (MWM) test

The Morris Water Maze (MWM) is a behavioral experiment designed to assess spatial learning and memory in rodents (rats, mice). During the experiment, the animals are required to swim and locate a hidden platform within a time limit of 1 min. The escape latency and the path taken by the animals to reach the platform are recorded. If the mouse is unable to locate the platform within 1 min, it should be placed on the platform for a duration of 10–30 s at the conclusion of each trial. Each mouse undergoes four trials per day over a period of 6 days. On the seventh day, a probe test is administered to assess memory consolidation, during which the platform is removed from the maze, and the mice are permitted to swim freely for 1 min. The number of times each mouse crosses the former platform location and the percentage of time spent exploring the quadrant zone should be monitored and recorded.

### 2.3 Novel object recognition (NOR) test

The Novel Object Recognition (NOR) test was conducted to evaluate memory related to novel object recognition. Initially, the mice were acclimated to the NOR apparatus (dimensions: 40 × 20 × 20 cm) for a duration of 5 min without the presence of any objects, after which they were returned to their home cages. During the acquisition phase, the mice were placed in the apparatus containing two identical cylindrical objects made of light brown wood (dimensions: height 10 cm, diameter 2 cm) and were permitted to explore freely for 5 min. On the subsequent day, one of the familiar objects was substituted with a novel object, specifically a wooden square pillar (dimensions: height 10 cm, width 2.5 cm, length 2 cm). The mice were then allowed to explore the apparatus freely once again. The sessions were video-recorded and analyzed for preference percentages using the following formula: [(time spent exploring the novel object or the familiar object)/(total time spent exploring both objects)] × 100%. Exploration was operationally defined as instances of touching and sniffing the objects with either the forepaws or the nose.

### 2.4 Y-maze test

In the Y-maze task, individual mice were positioned in one arm and permitted to explore freely for a duration of 5 min. The sequence of arm entries was digitally recorded. This process was documented using a camera and subsequently analyzed with Supermaze software (Xinruan Information Technology Co., Ltd., China). The sequence of arm entries was also visually observed. Spontaneous alternation was defined as the successive entry of the mice into all three arms in overlapping triplet sets. The alternation behavior percentage was calculated using the formula: [number of successive triplet sets (entries into three different arms consecutively)/(total number of arm entries - 2)] × 100%.

### 2.5 Immunofluorescence staining (IF)

Immunofluorescence staining of murine brain tissues was conducted following established protocols. In summary, brain tissues were sectioned to a thickness of 4 μm, rinsed with 0.01 M PBS, and incubated in a solution containing 5% bovine serum albumin and 0.25% Triton X-100. The tissue sections were subsequently washed with PBS at ambient temperature and incubated overnight with the primary antibody. On the following day, the samples were again washed with PBS and incubated for 2 h with the appropriate secondary antibodies at room temperature. The tissue sections were washed with PBS and subsequently stained with diamidino-2-phenylindole (DAPI, Beyotime, China). Imaging was performed using a fluorescence microscope (Carl Zeiss AG, Zeiss Axio Imager2).

### 2.6 Cell culture and cell treatments

HT22 cells were cultured in Dulbecco’s Modified Eagle’s Medium (DMEM, BasalMedia, Shanghai) supplemented with 10% fetal bovine serum and 1% penicillin-streptomycin solution, under humidified conditions (5% CO_2_) at 37°C. In the cell viability experiment, HT22 cells were seeded into a 96-well plate at a density of 5 × 10^3^ cells per well. For other cell experiments, HT22 cells were seeded into a 48-well plate at a density of 2.5 × 10^4^ cells per well. To investigate the effects of E4A and melatonin on D-gal-induced neuronal senescence, HT22 cells were categorized into five groups: (1) control group; (2) D-gal (200 mM) group; (3) D-gal + E4A (10 nM) group; (4) D-gal + MLT (50 μM) group; and (5) D-gal + E4A + MLT group. Additionally, to assess the impact of E4A and melatonin on neuron ferroptosis, HT22 cells were treated with 10 μM of Erastin (SC0224, Beyotime). The experimental groups were as follows: (1) control group; (2) Erastin group; (3) Erastin + E4A (10 nM) group; (4) Erastin + MLT (50 μM) group; and (5) Erastin + E4A+ MLT group. Furthermore, to evaluate the role of Nrf2 in mediating the therapeutic effects of E4A and melatonin in neuronal senescence, 5 μM of Nrf2 inhibitor AEM1 (HY-113848, Medchem Express) was administered independently for 2 hours prior to the treatment of D-gal (200 mM). The HT22 cells were then divided into six groups: (1) control group; (2) D-gal group; (3) D-gal + E4A (10 nM) group; (4) D-gal + E4A + AEM1 group; (5) D-gal + MLT (50 μM) group; and (6) D-gal + MLT + AEM1 group

### 2.7 Cell Viability Assay

HT22 cells were seeded into a 96-well plate at a density of 5 × 10^3^ cells per well and cultured for 24 h. Following drug treatment, cell viability was assessed using a CCK-8 assay kit (BS350B, Biosharp, Hefei, China). A 1:10 (v/v) dilution of CCK-8 solution in the culture medium was applied for 1 h at 37°C. Subsequently, absorbance was measured at 450 nm using a microplate reader (Bio-Tek, Winooski, VT, United States).

### 2.8 SA-β-gal activity

The cells were stained using an SA-β-gal staining kit (C0602, Beyotime) according to the manufacturer’s instructions. Specifically, cells were rinsed in PBS and fixed for 15 min. Following three additional washes in PBS (3 min per wash), the cells were stained in the working solution for 24–48 h. Positive cells, which appeared blue, were evaluated at a magnification of 250× using an MI52 light microscope (Guangzhou Mingmei Optoelectronic Technology Co., Ltd., Guangzhou, China). The resulting images were analyzed, and the cells were quantified utilizing ImageJ version 1.6 (NIH, Bethesda, MD, United States).

### 2.9 Quantification of GSH, MDA, and ROS Levels 

The levels of GSH and MDA in HT22 cells were quantified using their respective commercial assay kits (A006-2-1 for GSH, and A003-1 for MDA, Nanjing Jiancheng Bioengineering Institute, Nanjing, China). Intracellular ROS production was assessed by measuring the fluorescence intensity of 2,7-dichlorodihydrofluorescein diacetate (DCFH-DA) (MA0219, Meilunbio, China). HT22 cells were stained with 10 μM DCFH-DA in a dark, humidified chamber for 30 min at 37°C. Following PBS rinsing, fluorescence microscopy (Carl Zeiss AG, Axio Vert A1, Germany) was employed to capture images, and ROS levels were quantified using ImageJ software.

### 2.10 Western blot

Tissue and cell lysates, prepared by lysis in RIPA buffer supplemented with PMSF and a phosphatase inhibitor, were subjected to centrifugation at 14,000 ×g for 15 min at 4°C. Protein concentrations were determined utilizing a BCA assay kit (AL006–01, ACE Biotechnology Inc., Nanjing, China). Subsequently, 25 micrograms of protein were separated via 4%–12% SDS-PAGE and transferred onto PVDF membranes (Merck Millipore Ltd., Darmstadt, Germany). Following blocking with 3% Bovine Serum Albumin (BSA; GC305010, Servicebio Biotech), the blots were incubated overnight at 4°C with primary antibodies. The antibodies used, diluted in primary antibody diluents (P0023A, Beyotime), included: rabbit anti-phospho-ErbB4 (bs-3220R, Bioss; 1:1,000), rabbit anti-ErbB4 (AF6807, Beyotime; 1:1,000), rabbit anti-phospho-Akt1 (AF1546, Beyotime; 1:1,000), mouse anti-Akt1 (sc-377457, Santa Cruz Biotech; 1:1,000), rabbit anti-Nrf2 (AF7623, Beyotime; 1:1,000), rabbit anti-TFRC (AF8136, Beyotime; 1:1,000), rabbit anti-SLC7A11 (GB115276, Servicebio Biotech; 1:1,000), rabbit anti-GPX4 (AF7020, Beyotime; 1:1,000), rabbit anti-Lamin B1 (12987-1-AP, Proteintech, 1:5,000), rabbit anti-p53 (AF5258, Beyotime; 1:1,000), mouse anti-p21 (AP021, Beyotime; 1:1,000), rabbit anti-p16 (bs-23797R, Bioss Biotech, Beijing, China; 1:1,000), and rabbit anti-GAPDH (GB15002, Servicebio Biotech; 1:2000). Following four washes in TBST (5 min each), the membranes were incubated with HRP-conjugated goat anti-rabbit (bs-40295G, Bioss Biotech; 1:1,000) or goat anti-mouse (bs-40296G, Bioss Biotech; 1:1,000) antibodies, as appropriate, for 1 h. Subsequently, the membranes were washed with TBST four times (5 min per wash). The bands were visualized using a gel imaging system (Tanon-2500B, Tanon Biotech, Shanghai, China) and analyzed by densitometry using ImageJ.

### 2.11 Statistical analysis

All results are presented as mean ± standard deviation (SD). Data analysis was conducted using GraphPad Prism 9, employing both paired and unpaired t-tests. For comparisons involving multiple groups, one-way ANOVA followed by Tukey’s *post hoc* test was utilized. Statistical significance was defined as p < 0.05. The number of replicates is specified in the figure legends.

## 3 Results

### 3.1 ErbB4 receptor agonist can improve cognitive and spatial memory dysfunction in D-gal induced aging mice

**FIGURE 1:**
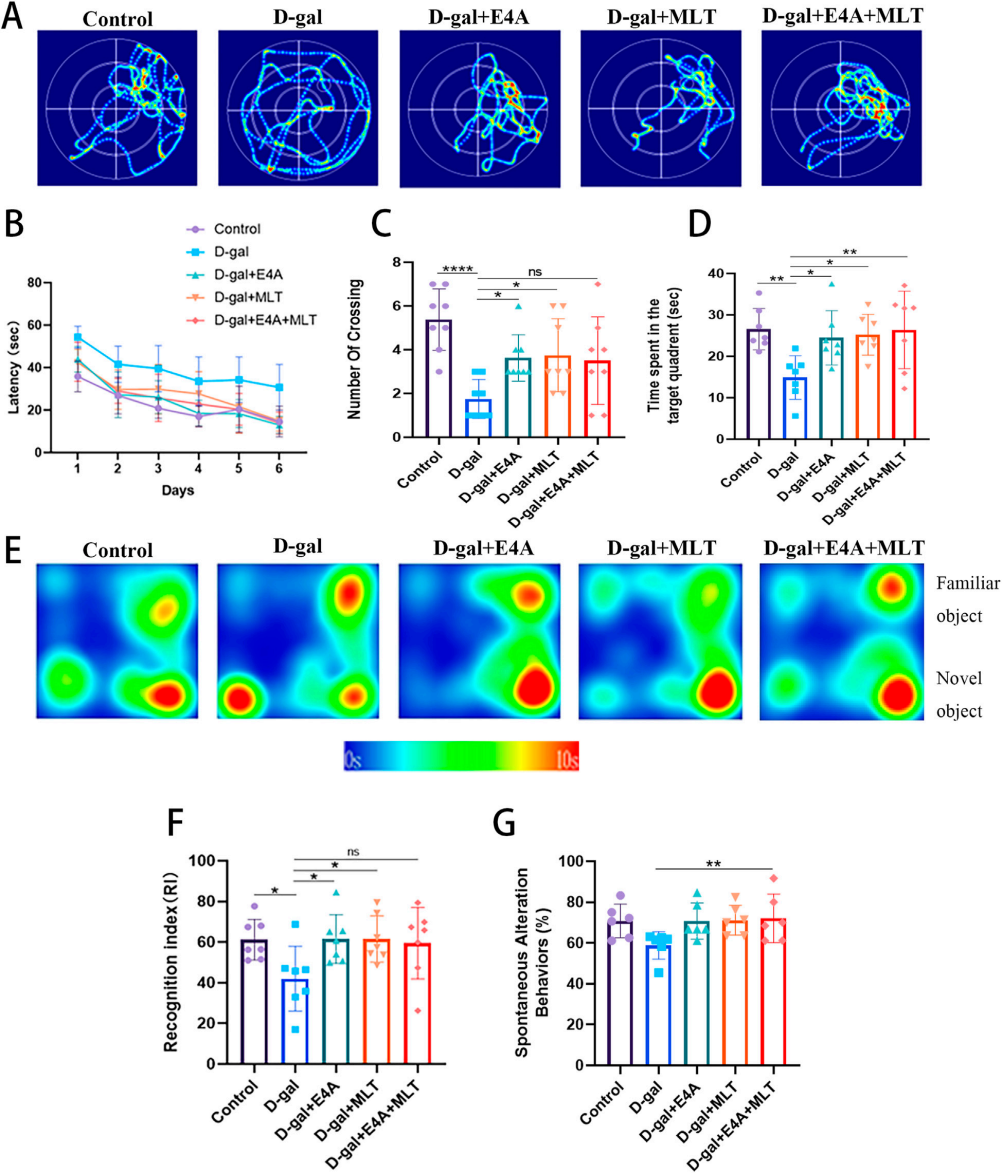
The effects of ErbB4 receptor agonist E4A and melatonin on cognitive and spatial memory deficits in D-gal-induced aging mice. **(A)** Representative swimming trajectories of mice subjected to different treatments in the Morris Water Maze (MWM) spatial probe test. **(B)** Escape latency across the five experimental groups. **(C)** Number of target crossings during the MWM probe test. **(D)** Time spent in the target quadrant during the MWM probe test. **(E)** Representative heat map showing interaction frequency with novel versus familiar objects. **(F)** Recognition index in the Novel Object Recognition test among the experimental groups. **(G)** Spontaneous alternation behaviors in the Y-maze test were assessed. Data were presented as mean ± SD (n = 6–8). Statistical significance was denoted as follows: *p < 0.05, **p < 0.01, ****p < 0.0001.

Spatial learning and memory in all animals were assessed using the Morris water maze test. During the training phase of this test, the mean escape latency was analyzed over a period of 6 days. The results indicated that chronic treatment with D-gal alone delayed the discovery of the hidden platform compared to the control group. Following treatment with E4A, melatonin, and a combination of the two drugs, there was an apparent reduction in escape latency (Figure 1B).

Subsequently, we employed a probe test to evaluate the spatial exploration and memory abilities of the mice. The number of platform crossings was significantly lower in the D-gal group (Figures 1A, C). Furthermore, the swimming time spent in the target quadrant by the D-gal group was less than that of the control mice (Figures 1A, D). Compared to the D-gal group, the drug treatment groups (E4A group, melatonin group, and combined treatment group) demonstrated a significant increase in target quadrant swimming time and platform crossing numbers (Figures 1A, C, D).

Recognition memory was assessed in mice using the NOR test (Figure 1E). The results from the NOR test indicated that the D-gal model mice exhibited a substantial reduction in recognition memory, with their recognition index being significantly lower than that of the control group. Following drug treatment, there was a notable increase in the recognition index (Figure 1F). In the Y-maze test, mice subjected to the treatment demonstrated a higher spontaneous alternation percentage compared to the D-gal group, with the combined treatment group achieving statistical significance (Figure 1G). In summary, D-gal-induced mice exhibit substantial memory and cognitive impairments, whereas treatment with ErbB4 receptor agonist yields effects comparable to melatonin, effectively enhancing the memory capabilities of D-gal-induced mice.

### 3.2 ErbB4 receptor agonist E4A can improve D-gal-induced aging of hippocampal neurons in mice

**FIGURE 2:**
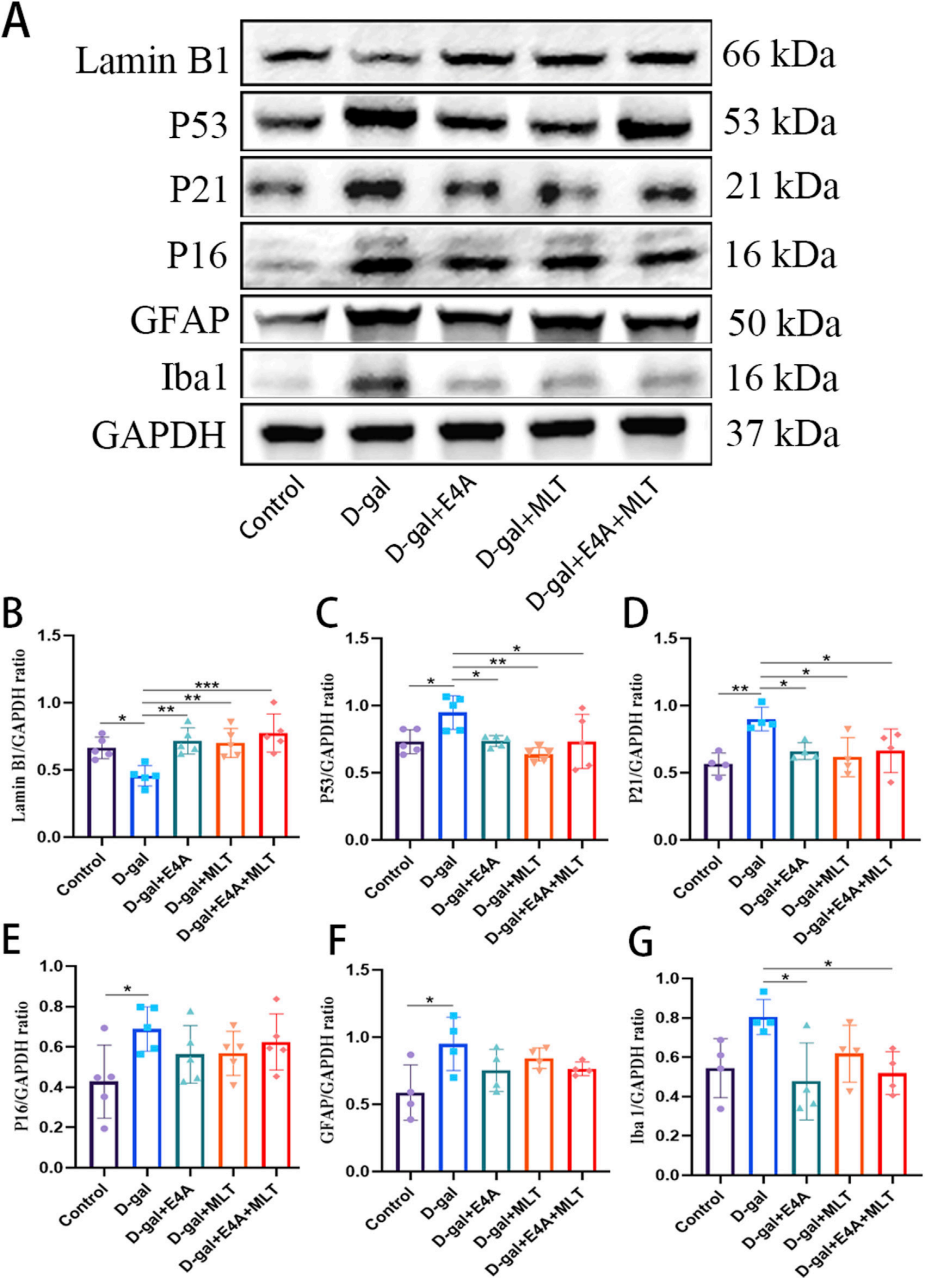
Effect of ErbB4 receptor agonist and melatonin can ameliorate D-gal-induced aging in hippocampus in mice. **(A)** Western blot analysis of Lamin B1, p53, p21, p16, GFAP, and Iba-1 protein expression levels in the hippocampus of mice. **(B–G)** Quantification of Lamin B1, p53, p21, p16, GFAP, and Iba-1 protein levels. Data are presented as mean ± SD, with n = 4–5. *p < 0.05, **p < 0.01, ***p < 0.001.

To explore the anti-aging effects of ErbB4 receptor agonist E4A and melatonin, we examined the expression of several key proteins associated with cellular aging using Western blot analysis. The results demonstrated that the protein levels of p53, p21, and p16 in the hippocampal tissues of D-gal-exposed mice were significantly upregulated compared to the control group (Figures 2A, C–E). The expression of Lamin B1 in aging mice was significantly reduced, indicating the onset of senescence (Figures 2A, B). However, treatment with E4A and melatonin effectively mitigated the aging effects induced by D-gal. Furthermore, no significant difference was observed in the anti-aging efficacy between the combination therapy group and the individual treatments (Figures 2A–E).

Accumulating evidence indicates that astrocytosis and microgliosis increase with age and are more pronounced in the D-gal-induced aging mouse model compared to control mice. We observed a significant increase in the protein expression level of GFAP in the hippocampus of the D-gal group compared to the control mice, which can be partially reversed by the individual application of E4A, melatonin, or their combined treatment, although no statistical significance was observed (Figures 2A, F). Similarly, we detected a significant increase in the protein expression level of Iba-1 in the hippocampus of the D-gal group compared to the control mice, which can be partially reversed by the individual application of E4A, melatonin, or their combined treatment, with statistical significance detected in the E4A and combined treatment group (Figures 2A, G). These findings suggest that ErbB4 receptor agonist, similar to melatonin, can ameliorate hippocampal neuron aging induced by D-gal in mice.

### 3.3 Ferroptosis inhibition by ErbB4 receptor agonist in D-gal-induced aging mice

**FIGURE 3:**
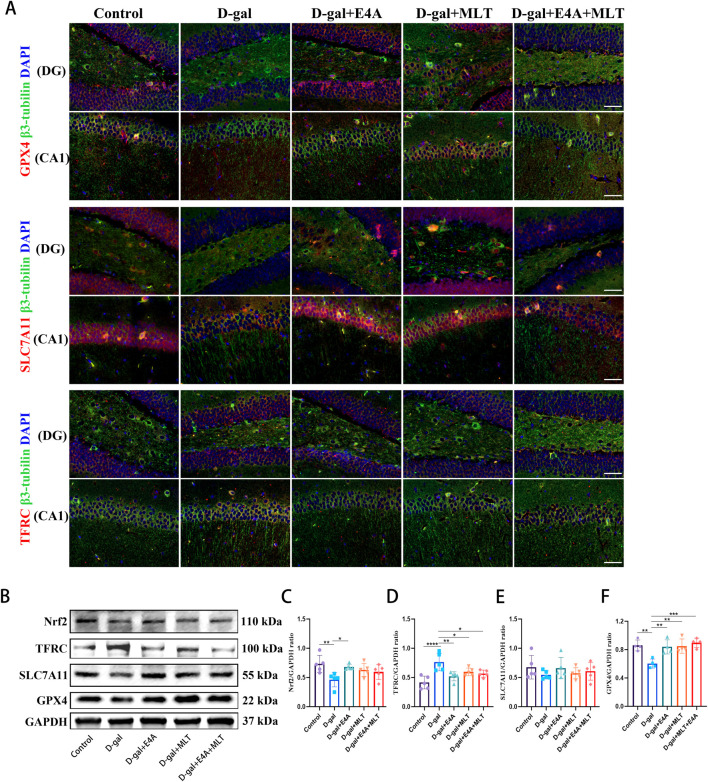
ErbB4 receptor agonist and melatonin inhibit ferroptosis in hippocampus in D-gal-induced aging mice. **(A)** Immunofluorescence analysis of GPX4-positive cells, SLC7A11-positive cells, TFRC-positive cells in the dentate gyrus (DG) and CA1 regions. Scale bar = 50 μm. **(B)** Western blot analysis of Nrf2, TFRC, SLC7A11, and GPX4 protein expression levels in the hippocampus of mice. **(C–F)** Quantification of Nrf2, TFRC, SLC7A11, and GPX4 protein levels. Data are presented as mean ± SD, with n = 4–5. *p < 0.05, **p < 0.01, ***p < 0.001, ****p < 0.0001.

To investigate whether the anti-aging effects of ErbB4 receptor agonist and melatonin are associated with the inhibition of ferroptosis, we examined key markers indicative of this process. Immunofluorescence analysis demonstrated a marked reduction in the expression of GPX4 and SLC7A11 within the DG and CA1 regions, alongside an upregulation of TFRC expression in comparison to the control group. Notably, administration of the ErbB4 receptor agonist and melatonin effectively reversed these alterations (Figure 3A). Subsequently, we utilized Western blot analysis to evaluate the expression of ferroptosis-related proteins, specifically Nrf2, TFRC, SLC7A11, and GPX4. The findings indicated that the expression levels of Nrf2, SLC7A11, and GPX4 were reduced, whereas TFRC expression was elevated in the D-gal group compared to the control group. However, treatment with ErbB4 receptor agonist and melatonin significantly reversed these alterations (Figures 3B–F). These results further corroborate that ErbB4 receptor agonist and melatonin mitigate the aging in hippocampus by modulating ferroptosis.

### 3.4 ErbB4 receptor agonist mitigates D-gal-induced ferroptosis in HT22 cells

**FIGURE 4:**
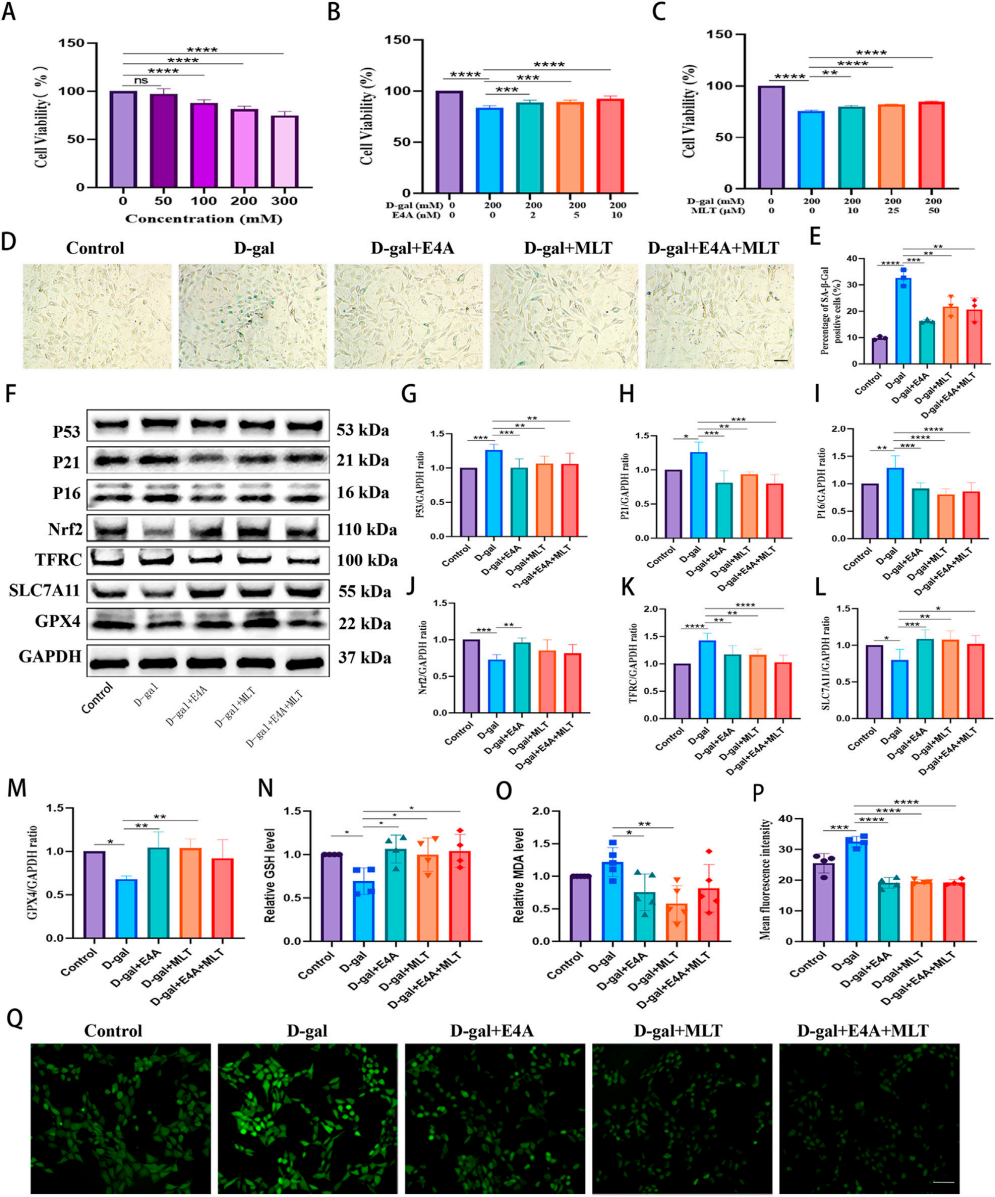
The mitigation of D-gal-induced ferroptosis in HT22 cells by ErbB4 receptor agonist and melatonin. **(A)** Cell viability post D-gal exposure, evaluated using the CCK-8 assay (n = 5). **(B)** Cell viability following co-treatment with D-gal and ErbB4 receptor agonist, assessed via the CCK-8 assay (n = 6). **(C)** Cell viability following co-treatment with D-gal and melatonin, determined by the CCK-8 assay (n = 6). **(D)** Visualization of cellular senescence through SA-β-gal staining. Scale bar = 100 μm. **(E)** Quantification of SA-β-gal staining intensities (n = 3). **(F)** Western blot analysis was conducted to assess the expression levels of senescence-associated markers and ferroptosis-related proteins in HT22 cells. **(G–M)** Quantification of Nrf2, TFRC, SLC7A11, GPX4, p53, p21, and p16 levels was performed, with normalization to GAPDH (n = 4–7). **(N)** Intracellular GSH levels (n = 4). **(O)** Intracellular MDA levels (n = 5). **(P)** Quantitative analysis of ROS levels was undertaken (n = 4). **(Q)** Intracellular ROS generation was measured using DCFH-DA, wherein ROS activity was reflected as green fluorescence intensity. Scale bar = 200 μm. Data are presented as mean ± SD. Statistical significance is indicated as follows: *p < 0.05, **p < 0.01, ***p < 0.001, ****p < 0.0001.

We then explored the relationship between ferroptosis and aging in HT22 cells activated by ErbB4 receptor agonist and melatonin. To assess the impact of D-gal on HT22 cells, we treated the cells with varying concentrations of D-gal for 24 h and measured cell viability using the CCK8 assay to establish a cellular aging model. The results indicate a dose-dependent decrease in cell viability with increasing concentrations of D-gal (Figure 4A). At a D-gal concentration of 200 mM, treatment of HT22 cells with varying concentrations of ErbB4 receptor agonist and melatonin for 24 h significantly enhances cell viability. This suggests that ErbB4 receptor agonist within the range of 2–10 nM and melatonin within the range of 10–50 mM exert a protective effect on D-gal-induced aged HT22 cells (Figures 4B, C).

The anti-aging effects of ErbB4 receptor agonist and melatonin on D-gal-induced aged HT22 cells were evaluated using β-galactosidase assays and Western blot. As illustrated in Figures 4D, E, the intensity of SA-β-gal significantly increased in correlation with the concentration of D-gal, compared to the vehicle control. Exposure to 200 mM D-gal clearly induced senescence in HT22 cells. However, the addition of ErbB4 receptor agonist and melatonin reversed these effects. The levels of senescence-associated protein markers, including p53, p16, and p21, were subsequently assessed in HT22 cells via Western blot, revealing an increase corresponding to the D-gal dosage. Nonetheless, following drug treatment, the expression of these aging-related proteins decreased significantly (Figures 4F–I), suggesting that ErbB4 receptor agonist and melatonin can inhibit D-gal-induced aging in HT22 cells.

To further investigate the protective effects of ErbB4 receptor agonist and melatonin on hippocampal neurons, we evaluated the levels of ferroptosis-related proteins, including Nrf2, TFRC, SLC7A11, and GPX4, in D-gal-induced HT22 cells (Figure 4F). Our analysis revealed notable changes in the expression of these ferroptosis-related proteins in D-gal-induced aged HT22 cells (Figure 4F). We observed alterations in the expression of ferroptosis-associated proteins in aged HT22 cells induced by D-gal. Specifically, the expression levels of ferroptosis-inhibitory proteins, including Nrf2, SLC7A11, and GPX4, were significantly downregulated, whereas the expression of the TFRC protein was upregulated. However, treatment with ErbB4 receptor agonist and melatonin effectively reversed these changes (Figures 4F, J–M). Subsequently, we evaluated intracellular lipid peroxidation levels. The data revealed an increase in MDA and ROS levels, coupled with a decrease in GSH levels in the D-gal group. However, treatment with the ErbB4 receptor agonist and melatonin significantly mitigated these effects, resulting in an elevation of GSH levels (Figure 4N) and a reduction in MDA (Figure 4O) and ROS levels (Figures 4P, Q). These findings indicate that the ErbB4 receptor agonist and melatonin can effectively counteract D-gal-induced cellular senescence, partially through the attenuation of ferroptosis in HT22 cells.

### 3.5 The administration of ErbB4 receptor agonist mitigated ferroptosis in erastin-exposed HT22 cells

**FIGURE 5:**
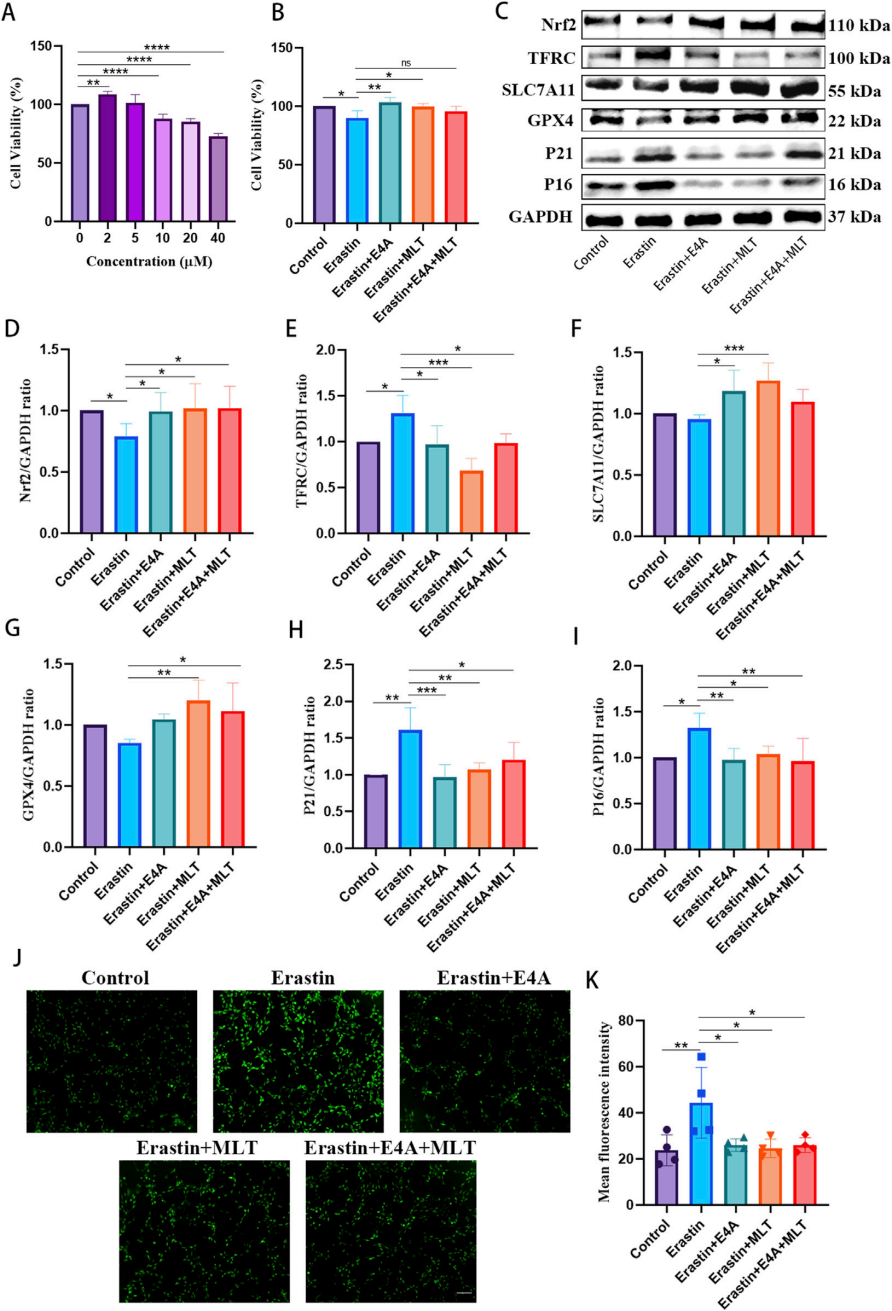
The effects of ErbB4 receptor agonist and melatonin treatment on ferroptosis in Erastin-exposed HT22 cells. **(A)** Cell viability of HT22 cells post-Erastin exposure was evaluated using the CCK-8 assay (n = 5). **(B)** Cell viability was evaluated following treatment with Erastin, cotreatment with either E4A or melatonin, or both, utilizing the CCK-8 assay (n = 4). **(C)** Western blot analysis was conducted to determine the expression levels of senescence-associated markers and ferroptosis-related proteins in HT22 cells. **(D–I)** Quantitative analysis of Nrf2, TFRC, SLC7A11, GPX4, p21, and p16 levels, normalized to GAPDH (n = 4–7). **(J)** Intracellular ROS generation was measured using DCFH-DA, with ROS activity indicated by green fluorescence. Scale bar = 200 μm. **(K)** The quantitative analysis of ROS levels was conducted (n = 4). The data are presented as mean ± SD. Statistical significance is indicated as follows: *p < 0.05, **p < 0.01, ***p < 0.001, ****p < 0.0001.

To validate the anti-ferroptotic effects of ErbB4 receptor agonist, we employed an Erastin-induced ferroptosis model in HT22 cells to further explore the relationship between cellular senescence and ferroptosis. As depicted in Figure 5A, cell viability decreased with increasing concentrations of Erastin. However, treatment with ErbB4 receptor agonist and melatonin significantly enhanced cell viability in Erastin-exposed HT22 cells (Figure 5B). In Erastin-exposed HT22 cells, Western blot analysis revealed a marked decrease in the expression of negative regulatory factors associated with ferroptosis, namely Nrf2, SLC7A11, and GPX4, while the expression of TFRC was significantly upregulated. Treatment with ErbB4 receptor agonist and melatonin effectively inhibited Erastin-induced ferroptosis (Figures 5C–G). Additionally, we assessed senescence-associated proteins via Western blot. The expression levels of P21 and P16 were significantly elevated in Erastin-exposed HT22 cells. However, drug treatment mitigated the increase in P21 and P16 protein levels (Figures 5C, H, I). DCFH-DA staining demonstrated that ErbB4 receptor agonists and melatonin treatment attenuated Erastin-induced lipid ROS production in HT22 cells (Figures 5J, K).

These findings indicate that ErbB4 receptor agonist and melatonin may inhibit Erastin-induced ferroptosis in HT22 cells. Furthermore, cells treated with Erastin exhibited characteristics of senescence, suggesting a potential link between cellular senescence and ferroptosis. Nevertheless, additional research is required to elucidate the precise mechanisms underlying this relationship.

### 3.6 ErbB4 receptor agonist inhibits D-gal-induced hippocampal neuronal aging both *in vivo* and *in vitro* by activating ErbB4 receptor and modulating the Akt/Nrf2 signaling pathway

**FIGURE 6:**
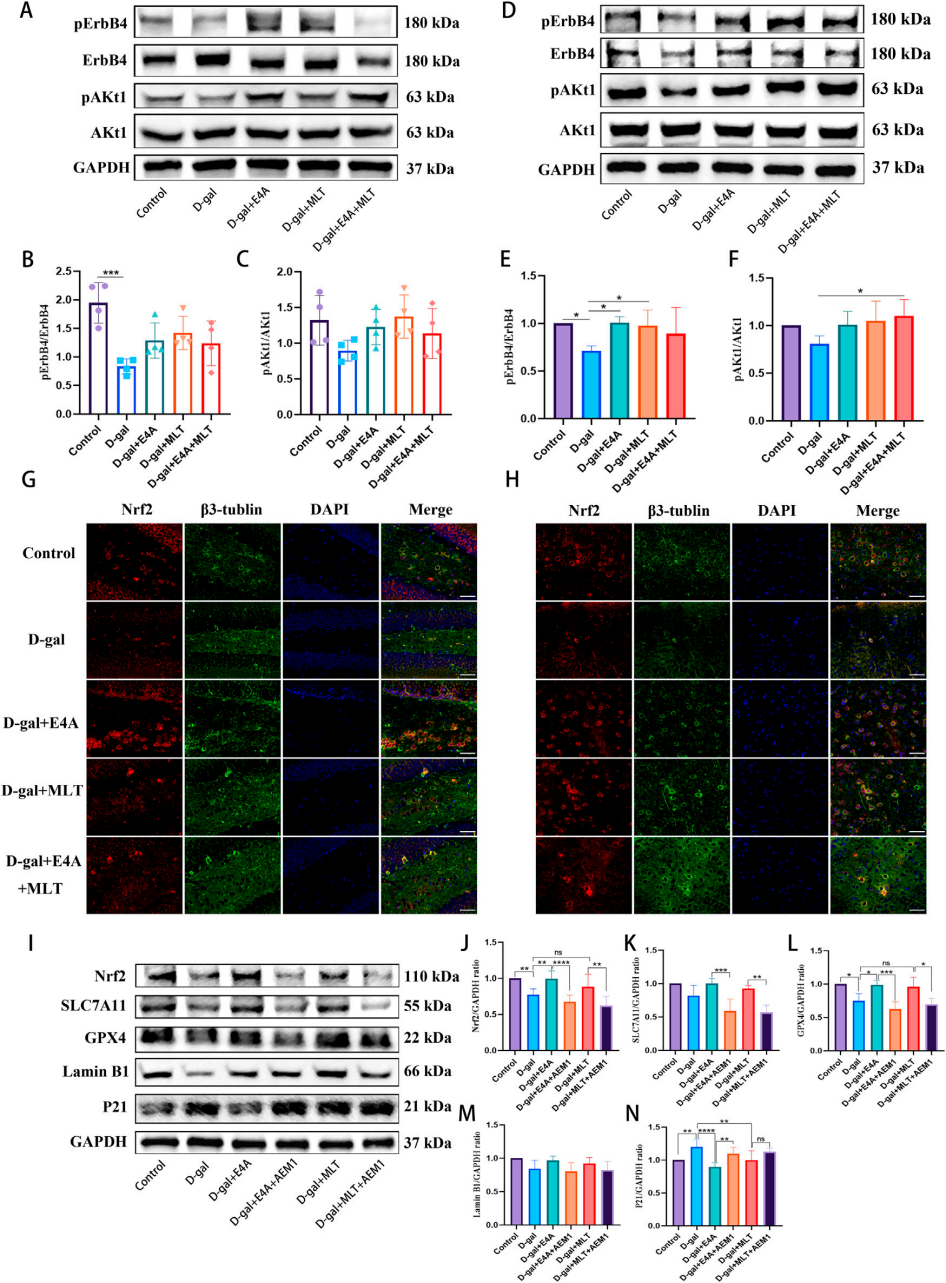
ErbB4 receptor agonist mitigates D-gal-induced hippocampal neuronal aging both *in vivo* and *in vitro* through the activation of ErbB4 receptors and modulation of the Akt/Nrf2 signaling pathway. **(A)** Western blot analysis of pErbB4, ErbB4, pAkt1, and Akt1 protein expression levels in the hippocampus of mice. **(B, C)** Quantification of pErbB4 and pAkt1 levels (n = 4). **(D)** Western blot analysis of pErbB4, ErbB4, pAkt1, and Akt1 protein expression levels in D-gal-induced HT22 cells. **(E, F)** Quantification of pErbB4 and pAkt1 levels (n = 4). **(G, H)** Immunofluorescence analysis of the Nrf2-positive cells in the DG region **(G)** and in the cortex region **(H)**. Scale bar = 50 μm. **(I)** Western blot analysis of Nrf2, SLC7A11, GPX4, Lamin B1 and P21 protein levels after treatment of Nrf2 inhibitor AEM1. **(J–N)** Quantitative analysis of Nrf2, SLC7A11, GPX4, Lamin B1 and P21 levels (n = 4–7). Data are presented as mean ± SD. Statistical significance is indicated as follows: *p < 0.05, **p < 0.01, ***p < 0.001, ****p < 0.0001.

To elucidate the specific mechanisms by which ErbB4 receptor agonist inhibit neuronal aging, we concurrently measured the protein expression levels of phosphorylated ErbB4 (pErbB4) and the downstream signaling molecule phosphorylated Akt1 (pAkt1) in both *in vivo* and *in vitro* models. Following D-gal induction, the levels of pErbB4 and pAkt1 were significantly downregulated both *in vivo* and *in vitro*. However, treatment with ErbB4 receptor agonist and melatonin resulted in the activation and subsequent upregulation of pErbB4 and pAkt1 expression (Figures 6A–F). Additionally, D-gal administration led to a downregulation of Nrf2 in the hippocampus and cortex of mice, indicating Nrf2 inactivation. Notably, the levels of Nrf2 in D-gal-induced aging mice were upregulated following treatment with ErbB4 receptor agonist and melatonin compared to the D-gal group (Figures 6G, H). The results indicate that ErbB4 receptor agonist and melatonin treatment can activate ErbB4 receptors and regulate the Akt/Nrf2 signaling pathway.

To further elucidate the role of the Akt/Nrf2 pathway in the effects mediated by ErbB4 receptor agonist and melatonin, we treated the cells with the Nrf2 inhibitor AEM1. After the treatment of AEM1, the expression of Nrf2 was significantly downregulated, indicating that AEM1 can effectively inhibit the expression of Nrf2. We also found that AEM1 obviously downregulated the level of SLC7A11 and GPX4 protein expressions. Furthermore, AEM1 inhibited the anti-aging effects of E4A and MLT (Figures 6I–N). These findings suggest that ErbB4 receptor agonist and melatonin can modulate ferroptosis through the Akt/Nrf2 pathway, thereby contributing to their neuroprotective effects.

## 4 Discussion

**FIGURE 7:**
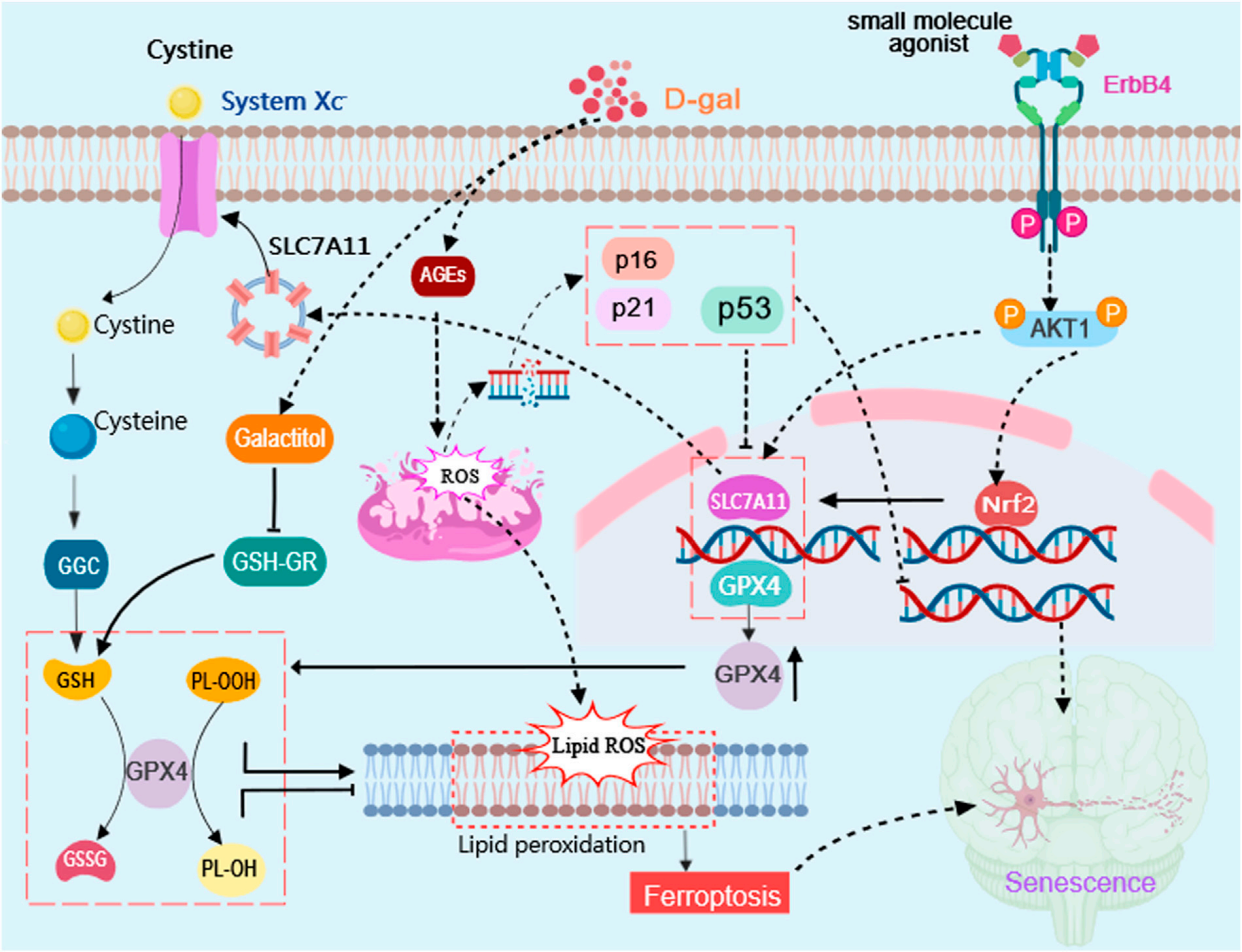
Schematic diagram illustrating the effects of targeted activation of ErbB4 receptor on neuronal aging via ferroptosis inhibition. Small molecule agonist (E4A)can activate ErbB4 receptors and regulate the Akt/Nrf2 signaling pathway to achieve neuroprotective effect in D-gal-induced neuronal aging. D-gal treatment increases the accumulation of advanced glycation end products (AGEs) which stimulates the production of reactive oxygen species (ROS) and increase the expression of senescence marker genes P53, P16, and P21. E4A can promote Akt1 phosphorylation and promote Nrf2 entrance into the nucleus, in which it involves in regulating the expression of ferroptosis suppressor gene, such as SLC7A11 and GPX4 to attenuate lipid peroxidation, which can inhibit cell ferroptosis and neuronal aging. The diagram was created with MedPeer (www.medpeer.cn).

The ErbB4 receptor tyrosine kinase plays a pivotal role as a critical regulator in various physiological and pathological processes (Ou et al., 2021). The significance of ErbB4 is underscored by its multifaceted involvement ranging from developmental processes to disease pathogenesis (Ou et al., 2021). The process of cellular senescence plays a significant role in the aging process as it leads to the accumulation of damaged cells. Targeting cellular senescence holds potential for preventing age-related neurodegenerative diseases. In this study, we aimed to investigate the impact of targeted activation of ErbB4 receptor on hippocampal neuronal senescence induced by D-gal (Figure 7).

Levels of NRG1 and ErbB4 were sustained throughout developmental stages and into adulthood. In addition, the longer-lived rodents had higher levels of NRG1 and ErbB4, suggesting that NRG1-ErbB4 signaling pivotally contribute to maximum lifespan potential (MLSP) via maintaining neuronal integrity in divergent species (Edrey et al., 2012). In the human subventricular zone (SVZ), transcript levels of ErbB4 peaked in neonates and declined with age, with the SVZ ErbB4 mRNA quantities significantly decreased by >85% to almost undetectable levels after the first year of life (Chong et al., 2008).

A progressive decline of ErbB4 expression was observed particularly in the substantia nigra pars compacta (SNpc), coinciding with a loss of the dopamine-synthesizing enzyme TH, implicating a role for diminished NRG/ErbB4 trophic support in dopamine-related neurodegenerative disorders of aging (Dickerson et al., 2009). Disruption of NRG1-ErbB4 signaling in the parvalbumin-positive interneurons partially contribute to the isoflurane-induced hippocampus-dependent cognitive impairment in aged mice exposed to isoflurane, which can be reversed by exogenous NRG1-β1 (Li et al., 2014). Additionally, impaired NRG1-ErbB4 signaling accelerates the impairment of neuronal networks underlying LTP in the hippocampal Schaffer-collateral pathway in 5xFAD mice, a phenomenon typically expected in aged wild-type mice (Seo et al., 2021). These data collectively presented the involvement of ErbB4 receptor signaling deficiency in neuronal senescence and neurodegeneration, indicating the therapeutic potential of targeted ErbB4 receptor activation in the treatment against neuronal senescence and further related neurodegenerative diseases.

NRG1 exhibits a pronounced inhibitory effect on stress-induced premature senescence by activating ErbB4 receptor in vascular cells (Shakeri et al., 2018). Additionally, enhanced ErbB4 signaling has been shown to facilitate the repair of infarcted adult mouse hearts and improve cardiac function by mitigating the progression of senescence and apoptosis in cardiac fibroblasts and aged mesenchymal stem cells (Liang et al., 2019; Shiraishi et al., 2022; Tao et al., 2015). Recent research reported NRG/ErbB signaling in delaying pituitary lactotroph cell senescence and enhancing PRL production via promoting TRPM8 expression under the modulation of melatonin (Lin et al., 2024; Zhang et al., 2024). To date, no studies have documented the therapeutic effects of targeted ErbB4 receptor activation in reversing neuronal senescence, both *in vitro* and *in vivo*. In this study, a small molecule ErbB4 receptor agonist, identified through virtual screening, was utilized. The activation of the ErbB4 receptor significantly ameliorated the behavioral deficits in D-gal-induced senescent mice. Furthermore, targeted activation of the ErbB4 receptor markedly reversed D-gal-induced neuronal senescence, indicating the potential of ErbB4 receptor activation as a therapeutic strategy for treating neuronal senescence and related neurodegenerative diseases.

In addition to reversing neuronal senescence, the agonist concurrently reduced the expression of ferroptosis promoters and enhanced the expression of ferroptosis inhibitors in both *in vitro* and *in vivo* models. We then specifically induced ferroptosis in mouse hippocampal HT22 cells, which was almost inhibited by the agonist treatment, further confirming that ErbB4 signaling may also function as a neuronal ferroptosis suppressor. Consistent with our findings, a recent study demonstrated that the ErbB4 receptor mediates the therapeutic effects of Ginsenoside Rd (G-Rd) by activating the PI3K/Akt/mTOR signaling pathway, thereby inhibiting cerebral ischemia/reperfusion injury (CIRI)-induced ferroptosis in cerebral vascular endothelial cells (Hu et al., 2024).

Accumulated evidence has supported the interplay between senescence and ferroptosis. Klotho, a senescence inhibitor, can eliminate D-gal-induced aging in H9C2 cells by increasing the expression of SLC7A11 and GPX4. Moreover, pifithrin-α, a P53-specific inhibitor, enhanced the expression of SLC7A11 and GPX4 (Xiong et al., 2023). The aging process has been linked to increased iron accumulation in neurons, thus promoting neurodegeneration through ferroptosis (Sfera et al., 2018). Excess intracellular iron has been shown to hasten the aging process by causing DNA damage and inhibiting genomic repair mechanisms referred to as ferrosenescence (Sfera et al., 2018). Oxidative stress, a significant downstream effect of ferroptosis, is also a key factor in cell senescence (Zhu et al., 2024). In the context of MPP^+^-induced ferroptosis in PC12 cells, Ferrostatin-1 (Fer-1) was found to downregulate p53 expression and upregulated SLC7A11 and GPX4 expression. Furthermore, inhibition of p53 eliminated cell senescence by upregulating the expression of SLC7A11 and GPX4, suggesting the involvement of the ferroptosis regulatory mechanism in MPP^+^-induced senescence (Li et al., 2021). We thus propose that ErbB4 receptor activation may treat neuronal senescence via inhibiting ferroptosis.

Recently, the pivotal role of Nrf2 in antagonizing ageing via the downregulation of ferroptosis has been widely reported (Fu et al., 2024; Li J. et al., 2023; Xiao et al., 2024; Xu et al., 2022). In an accelerated aging rat model induced by D-gal, a disturbance in anti-ferroptosis pathways was identified, leading to a concurrent decline in cognitive functions. Remarkably, the enhancement of Nrf2 expression mitigated this disturbance by reducing the expression of ferroptosis-related proteins, consequently ameliorating the observed cognitive impairments (Xiao et al., 2024). Eldecalcitol reduced intracellular ROS and MDA productions, elevated JC-1 aggregates, and upregulated expression of Nrf2 and GPX4, exhibiting osteoprotective effects in D-gal-induced aging ovariectomized (OVX) mice (Fu et al., 2024). Vitamin D (VD) has been reported to alleviate ferroptosis in aging hippocampal neurons by increasing GSH levels while reducing MDA and intracellular and mitochondrial ROS levels, which is mediated by the VDR/Nrf2/HO-1 signaling pathway (Li J. et al., 2023). Xu et al. (Xu et al., 2022) confirmed the essential role of ferroptosis in age-related osteoporosis, showing that VDR activation attenuated osteoblast ferroptosis via stimulating the Nrf2/GPX4 signaling pathway. In the present study, among the principal inhibitors of ferroptosis, the expression of the protein Nrf2 was most significantly altered in both the hippocampus and hippocampal neuronal cells following exposure to D-gal. However, this alteration was nearly normalized upon co-treatment with an ErbB4 agonist. These findings collectively suggest that the inhibition of neuronal senescence by the ErbB4 receptor agonist is largely mediated through the upregulation of Nrf2 expression.

Studies have shown that melatonin has dual effects of anti-ferroptosis and anti-aging, so it was selected as a positive control drug in this study. Melatonin improves stroke by inhibiting autophagy-dependent ferroptosis mediated by NCOA4 binding to FTH1 (Yu et al., 2024). Melatonin exerts its effects through the MT2 receptor, activating Nrf2 and downstream genes such as HO-1/NQO1 to inhibit ferroptosis in neuronal injury induced by subarachnoid hemorrhage, leading to improved neurological function in rats (Ma and Farny, 2023). Overexpression and activation of MT1 partly block ferroptosis in PD by preventing α-syn PFF-induced intracellular α-syn aggregation and enhancing the expression of the SIRT1/Nrf2/HO-1/GPX4 pathway and FTH1 protein (Lv et al., 2024). Furthermore, exogenous melatonin treatment demonstrates a protective effect against hypoxic-ischemic brain damage through the Akt/Nrf2/GPX4 pathway (Gou et al., 2020).

Our findings demonstrate that the targeted activation of the ErbB4 receptor effectively inhibits D-gal-induced neuronal senescence-associated and ferroptosis-related molecules in a manner akin to melatonin. This observation implies that these two agents may utilize comparable mechanisms to attenuate neuronal senescence by inhibiting ferroptosis. Nonetheless, the ErbB4 receptor agonist exhibits a greater reliance on the AKT signaling pathway to counteract ferroptosis in D-gal-induced senescent mice compared to melatonin. In comparison to conventional recombinant protein therapeutics, small molecule compounds exhibit superior permeability across the central nervous system, thereby enhancing their therapeutic efficacy in the treatment of neurodegenerative diseases. Furthermore, small molecule compounds are more cost-effective and hold significant promise for clinical translational applications (Li Q. et al., 2023).

The concurrent administration of an ErbB4 receptor agonist and melatonin did not enhance the inhibition of D-gal-induced senescence *in vitro* or *in vivo* beyond the effects observed with the individual administration of each compound. In addition to upregulating ErbB4 expression (Moshkdanian et al., 2017; Shafiei et al., 2023), it is supposed that melatonin may exert neuroprotective effects through its interaction with the ErbB4 receptor (Pankratova et al., 2018). It is possible that the ErbB4 agonist competes with melatonin for binding to the receptor, thereby diminishing any potential synergistic effect.

In summary, our data suggest that targeted activation of the ErbB4 receptor may be a viable strategy for treating neuronal senescence by inhibiting ferroptosis, thereby offering a potential therapeutic avenue for senescence-associated neurodegenerative diseases.

## Data Availability

The original contributions presented in the study are included in the article/supplementary material, further inquiries can be directed to the corresponding authors.

## References

[B1] Ahsan A. Hiniker S. M. Davis M. A. Lawrence T. S. Nyati M. K. (2009). Role of cell cycle in epidermal growth factor receptor inhibitor-mediated radiosensitization. Cancer Res. 69 (12), 5108–5114. 10.1158/0008-5472.CAN-09-0466 19509222 PMC2697971

[B2] Ashraf A. Jeandriens J. Parkes H. G. So P. W. (2020). Iron dyshomeostasis, lipid peroxidation and perturbed expression of cystine/glutamate antiporter in Alzheimer's disease: evidence of ferroptosis. Redox Biol. 32, 101494. 10.1016/j.redox.2020.101494 32199332 PMC7083890

[B3] Becker N. V. Scott J. W. Moniz M. H. Carlton E. F. Ayanian J. Z. (2022). Association of chronic disease with patient financial outcomes among commercially insured adults. JAMA Intern Med. 182 (10), 1044–1051. 10.1001/jamainternmed.2022.3687 35994265 PMC9396471

[B4] Bellinger F. P. Bellinger M. T. Seale L. A. Takemoto A. S. Raman A. V. Miki T. (2011). Glutathione peroxidase 4 is associated with neuromelanin in substantia nigra and dystrophic axons in putamen of Parkinson's brain. Mol. Neurodegener. 6 (1), 8. 10.1186/1750-1326-6-8 21255396 PMC3037910

[B5] Bishop N. A. Lu T. Yankner B. A. (2010). Neural mechanisms of ageing and cognitive decline. Nature 464 (7288), 529–535. 10.1038/nature08983 20336135 PMC2927852

[B6] Buonanno A. Fischbach G. D. (2001). Neuregulin and ErbB receptor signaling pathways in the nervous system. Curr. Opin. Neurobiol. 11 (3), 287–296. 10.1016/s0959-4388(00)00210-5 11399426

[B7] Chen D. Zhang T. Lee T. H. (2020). Cellular mechanisms of melatonin: insight from neurodegenerative diseases. Biomolecules 10 (8), 1158. 10.3390/biom10081158 32784556 PMC7464852

[B8] Chong V. Z. Webster M. J. Rothmond D. A. Weickert C. S. (2008). Specific developmental reductions in subventricular zone ErbB1 and ErbB4 mRNA in the human brain. Int. J. Dev. Neurosci. 26 (7), 791–803. 10.1016/j.ijdevneu.2008.06.004 18662768

[B9] Cook C. I. Yu B. P. (1998). Iron accumulation in aging: modulation by dietary restriction. Mech. Ageing Dev. 102 (1), 1–13. 10.1016/s0047-6374(98)00005-0 9663787

[B10] Dickerson J. W. Hemmerle A. M. Numan S. Lundgren K. H. Seroogy K. B. (2009). Decreased expression of ErbB4 and tyrosine hydroxylase mRNA and protein in the ventral midbrain of aged rats. Neuroscience 163 (1), 482–489. 10.1016/j.neuroscience.2009.06.008 19505538 PMC2755587

[B11] Dixon S. J. Lemberg K. M. Lamprecht M. R. Skouta R. Zaitsev E. M. Gleason C. E. (2012). Ferroptosis: an iron-dependent form of nonapoptotic cell death. Cell 149 (5), 1060–1072. 10.1016/j.cell.2012.03.042 22632970 PMC3367386

[B12] Edrey Y. H. Casper D. Huchon D. Mele J. Gelfond J. A. Kristan D. M. (2012). Sustained high levels of neuregulin-1 in the longest-lived rodents; a key determinant of rodent longevity. Aging Cell 11 (2), 213–222. 10.1111/j.1474-9726.2011.00772.x 22103690 PMC4399559

[B13] Fang S. J. Li P. Y. Wang C. M. Xin Y. Lu W. W. Zhang X. X. (2017). Inhibition of endoplasmic reticulum stress by neuregulin-1 protects against myocardial ischemia/reperfusion injury. Peptides 88, 196–207. 10.1016/j.peptides.2016.12.009 27993557

[B14] Friedmann Angeli J. P. Schneider M. Proneth B. Tyurina Y. Y. Tyurin V. A. Hammond V. J. (2014). Inactivation of the ferroptosis regulator Gpx4 triggers acute renal failure in mice. Nat. Cell Biol. 16 (12), 1180–1191. 10.1038/ncb3064 25402683 PMC4894846

[B15] Fu Y. F. Guo Y. X. Xia S. H. Zhou T. T. Zhao Y. C. Jia Z. H. (2024). Eldecalcitol protected osteocytes against ferroptosis of D-gal-induced senescent MLO-Y4 cells and ovariectomized mice. Exp. Gerontol. 189, 112408. 10.1016/j.exger.2024.112408 38521178

[B16] Gao Y. Z. Wu X. M. Zhou Z. Q. Liu P. M. Yang J. J. Ji M. H. (2023). Dysfunction of NRG1/ErbB4 signaling in the Hippocampus might mediate long-term memory decline after systemic inflammation. Mol. Neurobiol. 60 (6), 3210–3226. 10.1007/s12035-023-03278-y 36840846

[B17] Gou Z. Su X. Hu X. Zhou Y. Huang L. Fan Y. (2020). Melatonin improves hypoxic-ischemic brain damage through the Akt/Nrf2/Gpx4 signaling pathway. Brain Res. Bull. 163, 40–48. 10.1016/j.brainresbull.2020.07.011 32679060

[B18] Hambright W. S. Fonseca R. S. Chen L. Na R. Ran Q. (2017). Ablation of ferroptosis regulator glutathione peroxidase 4 in forebrain neurons promotes cognitive impairment and neurodegeneration. Redox Biol. 12, 8–17. 10.1016/j.redox.2017.01.021 28212525 PMC5312549

[B19] Herdy J. R. Traxler L. Agarwal R. K. Karbacher L. Schlachetzki J. C. M. Boehnke L. (2022). Increased post-mitotic senescence in aged human neurons is a pathological feature of Alzheimer's disease. Cell Stem Cell 29 (12), 1637–1652 e6. 10.1016/j.stem.2022.11.010 36459967 PMC10093780

[B20] Hu S. Fei Y. Jin C. Yao J. Ding H. Wang J. (2024). Ginsenoside Rd enhances blood-brain barrier integrity after cerebral ischemia/reperfusion by alleviating endothelial cells ferroptosis via activation of NRG1/ErbB4-mediated PI3K/Akt/mTOR signaling pathway. Neuropharmacology 251, 109929. 10.1016/j.neuropharm.2024.109929 38521230

[B21] Ishikawa S. Ishikawa F. (2020). Proteostasis failure and cellular senescence in long-term cultured postmitotic rat neurons. Aging Cell 19 (1), e13071. 10.1111/acel.13071 31762159 PMC6974705

[B22] Jurk D. Wang C. Miwa S. Maddick M. Korolchuk V. Tsolou A. (2012). Postmitotic neurons develop a p21-dependent senescence-like phenotype driven by a DNA damage response. Aging Cell 11 (6), 996–1004. 10.1111/j.1474-9726.2012.00870.x 22882466 PMC3533793

[B23] Li J. Cao Y. Xu J. Li J. Lv C. Gao Q. (2023a). Vitamin D improves cognitive impairment and alleviates ferroptosis via the Nrf2 signaling pathway in aging mice. Int. J. Mol. Sci. 24 (20), 15315. 10.3390/ijms242015315 37894993 PMC10607218

[B24] Li Q. Ma Z. Qin S. Zhao W. J. (2023b). Virtual screening-based drug development for the treatment of nervous system diseases. Curr. Neuropharmacol. 21 (12), 2447–2464. 10.2174/1570159X20666220830105350 36043797 PMC10616913

[B25] Li S. Wang M. Wang Y. Guo Y. Tao X. Wang X. (2021). p53-mediated ferroptosis is required for 1-methyl-4-phenylpyridinium-induced senescence of PC12 cells. Toxicol In Vitro 73, 105146. 10.1016/j.tiv.2021.105146 33737050

[B26] Li X. M. Su F. Ji M. H. Zhang G. F. Qiu L. L. Jia M. (2014). Disruption of hippocampal neuregulin 1-ErbB4 signaling contributes to the hippocampus-dependent cognitive impairment induced by isoflurane in aged mice. Anesthesiology 121 (1), 79–88. 10.1097/ALN.0000000000000191 24589481 PMC4062586

[B27] Li Y. Zhang J. Wan J. Liu A. Sun J. (2020). Melatonin regulates Aβ production/clearance balance and Aβ neurotoxicity: a potential therapeutic molecule for Alzheimer's disease. Biomed. Pharmacother. 132, 110887. 10.1016/j.biopha.2020.110887 33254429

[B28] Liang X. Ding Y. Lin F. Zhang Y. Zhou X. Meng Q. (2019). Overexpression of ERBB4 rejuvenates aged mesenchymal stem cells and enhances angiogenesis via PI3K/AKT and MAPK/ERK pathways. FASEB J. 33 (3), 4559–4570. 10.1096/fj.201801690R 30566395

[B29] Lin W. W. Ou G. Y. Dai H. F. Zhao W. J. (2024). Neuregulin 4 (Nrg4) cooperates with melatonin to regulate the PRL expression via ErbB4/Erk signaling pathway as a potential prolactin (PRL) regulator. J. Cell Biochem. 125 (5), e30551. 10.1002/jcb.30551 38465779

[B30] Lv Q. K. Tao K. X. Yao X. Y. Pang M. Z. Cao B. E. Liu C. F. (2024). Melatonin MT1 receptors regulate the Sirt1/Nrf2/Ho-1/Gpx4 pathway to prevent α-synuclein-induced ferroptosis in Parkinson's disease. J. Pineal Res. 76 (2), e12948. 10.1111/jpi.12948 38488331

[B31] Ma Y. Farny N. G. (2023). Connecting the dots: neuronal senescence, stress granules, and neurodegeneration. Gene 871, 147437. 10.1016/j.gene.2023.147437 37084987 PMC10205695

[B32] Mahoney-Sanchez L. Bouchaoui H. Ayton S. Devos D. Duce J. A. Devedjian J. C. (2021). Ferroptosis and its potential role in the physiopathology of Parkinson's Disease. Prog. Neurobiol. 196, 101890. 10.1016/j.pneurobio.2020.101890 32726602

[B33] Min S. S. An J. Lee J. H. Seol G. H. Im J. H. Kim H. S. (2011). Neuregulin-1 prevents amyloid β-induced impairment of long-term potentiation in hippocampal slices via ErbB4. Neurosci. Lett. 505 (1), 6–9. 10.1016/j.neulet.2011.05.246 21787838

[B34] Moshkdanian G. Moghani-Ghoroghi F. Pasbakhsh P. Nematollahi-Mahani S. N. Najafi A. Kashani S. R. (2017). Melatonin upregulates ErbB1 and ErbB4, two primary implantation receptors, in pre-implantation mouse embryos. Iran. J. Basic Med. Sci. 20 (6), 655–661. 10.22038/IJBMS.2017.8833 28868121 PMC5569443

[B35] Ou G. Y. Lin W. W. Zhao W. J. (2021). Neuregulins in neurodegenerative diseases. Front. Aging Neurosci. 13, 662474. 10.3389/fnagi.2021.662474 33897409 PMC8064692

[B36] Pankratova S. Klingelhofer J. Dmytriyeva O. Owczarek S. Renziehausen A. Syed N. (2018). The S100A4 protein signals through the ErbB4 receptor to promote neuronal survival. Theranostics 8 (14), 3977–3990. 10.7150/thno.22274 30083275 PMC6071530

[B37] Seo H. J. Park J. E. Choi S. M. Kim T. Cho S. H. Lee K. H. (2021). Inhibitory neural network's impairments at hippocampal CA1 LTP in an aged transgenic mouse model of Alzheimer's disease. Int. J. Mol. Sci. 22 (2), 698. 10.3390/ijms22020698 33445678 PMC7828160

[B38] Sfera A. Bullock K. Price A. Inderias L. Osorio C. (2018). Ferrosenescence: the iron age of neurodegeneration? Mech. Ageing Dev. 174, 63–75. 10.1016/j.mad.2017.11.012 29180225

[B39] Shafiei G. Moghani-Ghoroghi F. Miyan J. Almasi M. Kashani I. R. Nikzad H. (2023). Melatonin protects against visible light-induced oxidative stress and promotes the implantation potential of mouse blastocyst *in vitro* . Res. Vet. Sci. 155, 29–35. 10.1016/j.rvsc.2022.12.003 36610243

[B40] Shakeri H. Gevaert A. B. Schrijvers D. M. De Meyer G. R. Y. De Keulenaer G. W. Guns P. D. F. (2018). Neuregulin-1 attenuates stress-induced vascular senescence. Cardiovasc Res. 114 (7), 1041–1051. 10.1093/cvr/cvy059 29528383

[B41] Shinde P. Vidyasagar N. Dhulap S. Dhulap A. Hirwani R. (2015). Natural products based P-glycoprotein activators for improved β-amyloid clearance in Alzheimer's disease: an *in silico* approach. Cent. Nerv. Syst. Agents Med. Chem. 16 (1), 50–59. 10.2174/1871524915666150826092152 26306632

[B42] Shiraishi M. Yamaguchi A. Suzuki K. (2022). Nrg1/ErbB signaling-mediated regulation of fibrosis after myocardial infarction. FASEB J. 36 (2), e22150. 10.1096/fj.202101428RR 34997943

[B43] Si Z. Sun L. Wang X. (2021). Evidence and perspectives of cell senescence in neurodegenerative diseases. Biomed. Pharmacother. 137, 111327. 10.1016/j.biopha.2021.111327 33545662

[B44] Tao L. Bei Y. Zhang H. Xiao J. Li X. (2015). Exercise for the heart: signaling pathways. Oncotarget 6 (25), 20773–20784. 10.18632/oncotarget.4770 26318584 PMC4673228

[B45] Tian Y. Tian Y. Yuan Z. Zeng Y. Wang S. Fan X. (2022). Iron metabolism in aging and age-related diseases. Int. J. Mol. Sci. 23 (7), 3612. 10.3390/ijms23073612 35408967 PMC8998315

[B46] Wang T. Tomas D. Perera N. D. Cuic B. Luikinga S. Viden A. (2022). Ferroptosis mediates selective motor neuron death in amyotrophic lateral sclerosis. Cell Death Differ. 29 (6), 1187–1198. 10.1038/s41418-021-00910-z 34857917 PMC9177596

[B47] Wang X. Wang Z. Cao J. Dong Y. Chen Y. (2021). Melatonin alleviates acute sleep deprivation-induced memory loss in mice by suppressing hippocampal ferroptosis. Front. Pharmacol. 12, 708645. 10.3389/fphar.2021.708645 34335271 PMC8322577

[B48] Wang Y. Lv M. N. Zhao W. J. (2023). Research on ferroptosis as a therapeutic target for the treatment of neurodegenerative diseases. Ageing Res. Rev. 91, 102035. 10.1016/j.arr.2023.102035 37619619

[B49] Xiao L. Wen H. Peng S. Chen B. Tang B. Liu B. (2024). Polygonatum polysaccharide ameliorates D-galactose-induced cognitive dysfunction in aging rats by inhibiting ferroptosis through activation of Nrf2. Neurosci. Lett. 836, 137873. 10.1016/j.neulet.2024.137873 38871020

[B50] Xiong X. Wang G. Wang Y. Zhang T. Bao Y. Wang K. (2023). Klotho protects against aged myocardial cells by attenuating ferroptosis. Exp. Gerontol. 175, 112157. 10.1016/j.exger.2023.112157 36990131

[B51] Xu P. Lin B. Deng X. Huang K. Zhang Y. Wang N. (2022). VDR activation attenuates osteoblastic ferroptosis and senescence by stimulating the Nrf2/GPX4 pathway in age-related osteoporosis. Free Radic. Biol. Med. 193 (Pt 2), 720–735. 10.1016/j.freeradbiomed.2022.11.013 36402439

[B52] Yu X. Wang S. Wang X. Li Y. Dai Z. (2024). Melatonin improves stroke by inhibiting autophagy-dependent ferroptosis mediated by NCOA4 binding to FTH1. Exp. Neurol. 379, 114868. 10.1016/j.expneurol.2024.114868 38901754

[B53] Zhang W. Dao J. J. Li Q. Liu C. Qiao C. M. Cui C. (2024). Neuregulin 1 mitigated prolactin deficiency through enhancing TRPM8 signaling under the influence of melatonin in senescent pituitary lactotrophs. Int. J. Biol. Macromol. 275 (Pt 1), 133659. 10.1016/j.ijbiomac.2024.133659 38969045

[B54] Zhu J. Chen H. Wu J. Li S. Lin W. Wang N. (2024). Ferroptosis in glaucoma: a promising avenue for therapy. Adv. Biol. (Weinh). 8 (5), e2300530. 10.1002/adbi.202300530 38411382

